# Do Human Milk Concentrations of Persistent Organic Chemicals Really Decline During Lactation? Chemical Concentrations During Lactation and Milk/Serum Partitioning

**DOI:** 10.1289/ehp.0900876

**Published:** 2009-06-15

**Authors:** Judy S. LaKind, Cheston M. Berlin, Andreas Sjödin, Wayman Turner, Richard Y. Wang, Larry L. Needham, Ian M. Paul, Jennifer L. Stokes, Daniel Q. Naiman, Donald G. Patterson

**Affiliations:** 1 LaKind Associates, LLC, Catonsville, Maryland, USA; 2 Department of Pediatrics, Milton S. Hershey Medical Center, Penn State College of Medicine, Hershey, Pennsylvania, USA; 3 Department of Epidemiology and Preventive Medicine, University of Maryland School of Medicine, Baltimore, Maryland, USA; 4 Division of Laboratory Sciences, National Center for Environmental Health, Centers for Disease Control and Prevention, Atlanta, Georgia, USA; 5 Department of Applied Mathematics and Statistics, The Johns Hopkins University, Baltimore, Maryland, USA; 6 EnviroSolutions Consulting, Inc., Jasper, Georgia, USA

**Keywords:** blood, breast milk, depuration, dioxins, elimination kinetics, infant exposure, partitioning, PBDEs, PCBs, pesticides

## Abstract

**Background:**

Conventional wisdom regarding exposures to persistent organic chemicals via breast-feeding assumes that concentrations decline over the course of lactation and that the mother’s body burden reflects her cumulative lifetime exposure. Two important implications stemming from these lines of thought are, first, that assessments of early childhood exposures should incorporate decreasing breast milk concentrations over lactation; and, second, that there is little a breast-feeding mother can do to reduce her infant’s exposures via breast-feeding because of the cumulative nature of these chemicals.

**Objectives:**

We examined rates of elimination and milk/serum partition coefficients for several groups of persistent organic chemicals.

**Methods:**

We collected simultaneous milk and blood samples of 10 women at two times postpartum and additional milk samples without matching blood samples.

**Results:**

Contrary to earlier research, we found that lipid-adjusted concentrations of polybrominated diphenyl ethers, polychlorinated biphenyls, polychlorinated dibenzo-*p*-dioxins and furans, and organochlorine pesticides in serum and milk do not consistently decrease during lactation and can increase for some women. Published research has also suggested an approximate 1:1 milk/serum relationship (lipid adjusted) on a population basis for 2,3,7,8-tetrachlorodibenzo-*p*-dioxin; however, our results suggest a more complex relationship for persistent, lipophilic chemicals with the milk/serum relationship dependent on chemical class.

**Conclusions:**

Decreases in concentration of lipophilic chemicals on a lipid-adjusted basis during lactation should no longer be assumed. Thus, the concept of pumping and discarding early milk as means of reducing infant exposure is not supported. The hypothesis that persistent lipophilic chemicals, on a lipid-adjusted basis, have consistent concentrations across matrices is likely too simplistic.

Exposures to persistent organic compounds and resulting health effects have been the subject of many scientific papers and global treaties. Of special concern are the potential effects of these chemicals on the fetus and infant. The ability to assess infant exposures to environmental chemicals via breast-feeding is crucial for developing a complete picture of early-life exposures to those chemicals. Conventional wisdom regarding lactational exposures to persistent organic chemicals presumes a decline in concentrations over the course of lactation [U.S. Environmental Protection Agency (EPA) 2003] and that the origin of those chemicals lay in a mother’s cumulative exposure over her lifetime (Miyata et al. 2006). Two important implications stemming from these lines of thought are that assessments of childhood exposures during the first year(s) of life should incorporate decreasing breast milk concentrations over the course of lactation and that there is little a breast-feeding mother can currently do to reduce her infant’s chemical exposures via breast-feeding.

The supposition that elimination of persistent organic chemicals via breast-feeding results in a decrease in breast milk concentrations during lactation derives from research that has measured levels of various chemicals in breast milk at different times postpartum. In fact, much of the past literature on this topic has shown relatively consistent results: levels decreasing over the course of lactation, albeit with different studies reporting different rates of elimination (LaKind et al. 2001). It has been suggested that about 60% of lipids in breast milk originate from the mother’s adipose tissue (Hachey et al. 1987), generally lending support to the concept that the predominant origin of lipophilic persistent chemicals in milk could be via remobilization of those chemicals stored in the mother’s adipose tissue. Consequently, as a woman breast-feeds, her stores of lipids and lipid-associated environmental chemicals are mobilized, and her accumulated levels would therefore decrease as the chemicals are excreted with breast milk. In fact, it seemed that the key piece of missing information for robust exposure assessments of the breast-fed infant was the quantification of chemical-specific rates of decrease in concentration. In the absence of that information, models of infant exposures via breast-feeding have made assumptions about rates of decrease in breast milk concentrations over time (e.g., 50% over 6 months) (LaKind et al. 2000).

For a complete assessment of infant exposures, in addition to being able to quantify changes in concentration over lactation, information on the initial concentrations of chemicals in breast milk is needed. Surveillance biomonitoring using human milk is convenient and gives information on levels of chemicals in the mother and infant exposures, but can also heighten concerns that breast-feeding may be unsafe. In addition, the availability of laboratories capable of performing analysis of breast milk for environmental chemicals is limited. Thus, if another biological matrix could be used as a surrogate for breast milk, then valuable exposure information would be obtained without negatively affecting breast-feeding rates or requiring increased laboratory capacity. A multitude of studies have examined blood levels of lipophilic persistent chemicals, including the National Health and Nutrition Examination Survey, a nationwide study conducted every 2 years in the United States [Centers for Disease Control and Prevention (CDC) 2005]. The determination of milk:serum partitioning coefficients that are stable over the duration of lactation could allow for the use of blood levels as a surrogate for estimating infant exposures from mother’s milk. Although a constant relationship for chemical concentrations in human tissues and fluids on a lipid-adjusted basis has been hypothesized based on research into lipid-adjusted serum:adipose tissue ratios for 2,3,7,8-tetrachlorodibenzo-*p*-dioxin (Patterson et al. 1988) and an initial review of earlier data suggested that on a population basis, breast milk lipid concentrations of persistent lipophilic compounds generally reflect blood lipid levels, albeit with significant variations in these ratios among individuals (Aylward et al. 2003), a more complex chemical- and tissue-specific relationship is emerging (Wittsiepe et al. 2007).

In this study, we sought to quantify rates of elimination and milk:serum partition coefficients for several groups of persistent organic chemicals by collecting simultaneous milk and blood samples from women at two times postpartum and additional milk samples without matching blood samples. This is the first study to provide data based on simultaneous sampling of breast milk and blood at separate times during lactation. We analyzed serum and milk for 17 polychlorinated dibenzo-*p*-dioxins and furans (PCDDs/PCDFs), 36 polychlorinated biphenyls (PCBs), 9 persistent chlorinated organic pesticides, and 10 polybrominated diphenyl ethers (PBDEs).

## Methods

The study was conducted with the approval of the Milton S. Hershey Medical Center Human Subjects Protection Office and complied with all applicable requirements of U.S. regulations. The CDC Institutional Review Board relied on that review under a cooperative research agreement. All study subjects gave informed consent.

### Study participants

A convenience cohort of 10 study participants was drawn from the population of women seeking prenatal care and women whose children receive pediatric care at the Penn State College of Medicine. Enrollment guidance was generally in accordance with the World Health Organization (WHO) guidelines for the study of persistent, organic compounds in human milk (WHO 2007); however, exclusive breast-feeding was not required for this investigation because of the limited number of women in the United States who exclusively breast-feed for extended periods of time. In addition, participants were eligible for inclusion if they had lived in the Lancaster County/Harrisburg area for the previous 3 (as opposed to 10) years.

Questionnaires were administered at the beginning and end of each mother’s participation in the study, with personal, demographic, lifestyle, and dietary information obtained. In addition, at the time of maternal milk and blood collection, we collected data on each infant’s age, weight, and history of intake of infant formula or other foods.

### Maternal milk and serum sampling

Participants washed their hands and breast prior to manually expressing the sample, with warm water and disposable breast pads used for cleaning, if desired. Milk was expressed into clean containers that were prescreened for cross-contaminants, including the analytes of interest. Participants provided milk samples at 1, 2, and 3 months postpartum and at the cessation of lactation. Approximately 30 mL (1 oz) milk per sampling collection event per mother was collected.

Blood was sampled by a trained phlebotomist at the same time (within 1 hr) of the milk sampling during the first and last milk donations. Approximately 15 mL serum was needed for the analytical component of this research, necessitating the collection of five tubes of 10 mL whole blood each. Serum from clotted blood was separated by centrifugation and stored in amber glass vials. Milk and serum specimens were stored frozen until they were shipped on dry ice via next-day delivery to the CDC. On arrival at the CDC, milk and blood specimens were stored at −70°C until analysis.

### Analytical method

Serum samples (2 mL) were fortified with ^13^C-labeled internal standards and formic acid and water for protein denaturation and diluted using a Gilson 215 liquid handler (Gilson Inc., Middleton, WI) for automation (Sjödin et al. 2004a). The samples were extracted by solid phase extraction (SPE) using the Rapid Trace modular SPE work station (Caliper Life Sciences, Hopkinton, MA) for automation. Removal of coextracted lipids was performed on a silica:silica/sulfuric acid column using the modular SPE work station for automation. Milk samples (1 mL) were processed using solid phase dispersion on Hydromatrix (Varian Inc., Walnut Creek, CA) and automatic fortification with ^13^C-labeled internal standards using the liquid handler (Sjödin et al. 2004b). The samples were then dried using pressurized nitrogen (8 kPa) and extracted by elution with dichloromethane using the modular SPE workstation for automation. The lipid concentration of the entire sample was determined gravimetrically using an AX105 Delta Range analytical balance (Mettler Toledo, Columbus, OH). Removal of coextracted lipids was performed using the same method as for serum.

Analytical determination of serum and milk PBDEs, PCBs, and organochlorine pesticides was performed by gas chromatography-isotope dilution high-resolution mass spectrometry (GC-IDHRMS) employing a MAT95XP (ThermoFinnigan MAT, Bremen, Germany) instrument. The serum lipid concentration was determined using commercially available test kits from Roche Diagnostics Corp. (Indianapolis, IN) for the quantitative determination of total triglycerides (product no. 011002803-0600) and total cholesterol (product no. 011573303-0600) (Phillips et al. 1989). Final determinations were made on a Hitachi 912 Chemistry Analyzer (Hitachi, Tokyo, Japan). Total serum lipid concentration was estimated from the concentration of total cholesterol and triglycerides. Every analytical run contained 24 unknown samples, three blanks and three quality control (QC) samples. The CV for the serum method is approximately 2–4% for QCs spiked at 500 pg/mL serum. The CV for the milk method is approximately 10% (the analysis of QC samples spiked at 500 pg/mL milk corresponds to approximately 10 ng/g lipid; hence, the QC samples were spiked at an environmentally relevant concentration).

For PCDDs/Fs and coplanar PCBs, serum (10 g) or milk samples (5 g) were spiked with ^13^C internal standards. We calculated serum total lipids using an enzymatic summation method (Phillips et al. 1989). Milk samples were extracted and lipid was determined gravimetrically. Serum and milk sample extracts were diluted with hexane, and the cleanup procedure followed the method of Turner et al. (1997). The concentrations of PCDDs/Fs and coplanar PCBs in serum and milk were determined by GC-IDHRMS (Patterson et al. 1987).

### Data analysis

#### Depuration

For a given chemical, to determine whether there was systematic evidence of a decrease (or increase) in serum concentration over time, we used a paired two-sample sign test. For seven of the study participants, two serum samples taken approximately 30 days postpartum and at the end of lactation (56–280 days postpartum) were available. The test statistic was the number of mothers of the seven whose serum concentrations decreased from the initial sampling to final sampling. Similarly, for testing the hypothesis of an increase in serum concentration, an analogous count was used. The test statistics for increases and decreases in concentration do not necessarily sum to seven, because in some cases the concentration for a given chemical did not change. Concentrations below the method detection limit for the chemical were excluded from the analysis.

For the milk data, we used the samples collected 1 and 3 months postpartum for analysis to maintain consistency of the time period examined. Nine of the 10 participants breast-fed for the 3-month time period. An analysis similar to that described for serum was performed.

Milk:serum partitioning.

We focused on milk:serum ratios from samples collected 1 month postpartum, as end-of-study collection times varied according to the participant’s duration of lactation. In addition, because results near the limit of detection (LOD) are subject to greater uncertainty, we limited the analyses to chemicals that were detected in all participants for serum and milk [bromodiphenyl ether (BDE) 47 and 100; hexachlorobenzene (HCB); *trans*-nonachlor; 1,1-dichloro-2,2-bis(*p*-chloro-phenyl)ethylene (*p,p′*-DDE); 1,1,1-trichloro-2,2-bis(*p*-chlorophenyl)ethane (*p,p′*-DDT); PCBs 74, 99, 118, 126, 138–158, 146, 153, 170, 180, and 187; 1,2,3,6,7,8-hexachloro-dibenzo-*p*-dioxin (HxCDD); 1,2,3,4,6,7,8-heptachlorodibenzo-*p*-dioxin (HpCDD); octachlorodibenzo-*p*-dioxin (OCDD); and 1,2,3,4,6,7,8-heptachlorodibenzofuran (HpCDF)]. For the 20 chemicals, we used least squares to fit a line through each set of 10 milk:serum pairs (from each of the 10 participants) and then used these fits to test whether the intercept was significant. Only one intercept was significant (BDE-100, *p* = 0.02). Therefore, we fit lines without intercepts for each chemical as


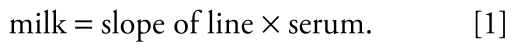


In other words, the slope of the line can be interpreted as an estimate of the milk:serum ratio. We also used a line fit on a log scale, that is,


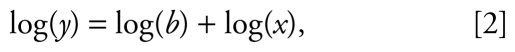


and then estimated log(*b*) to be the average of the difference log(*y*) – log(*x*) (i.e., taking an estimated *b* to be the geometric mean of all of the ratios *y*/*x*).

## Results

Ten mothers participated in the study. [See Supplemental Material, Table 1, for demographic and personal data; available online (doi:10.1289/ehp.0900876.S1 via http://dx.doi.org/)]. Seventeen serum samples and 35 milk samples were collected. Information on the timing of collection of milk and blood for the 10 participants is given in Table 1.

Median concentrations in milk and serum lipids and percent detects for all chemicals detected in at least 50% of serum and milk samples are given in Table 2. Measurements below the LOD were set equal to reported LOD for each sample. Units are nanograms per gram lipid except for PCDDs/Fs and PCBs 126 and 169 (picograms per gram lipid).

### Depuration

#### Serum

First and last serum samples were used to determine whether changes in concentrations of PCDDs/Fs, PCBs, PBDEs, or organochlorine pesticides were observed. For some of the mothers a time period of almost 1 year was available (Table 1); we therefore assumed that any potential long-term decreases (or increases) in serum concentrations would be observable. None of the chemical concentrations decreased over the course of lactation for all mothers. Specifically, when a decrease in serum concentration of a chemical was observed, it was never observed for more than five of the mothers (*p* = 0.227). However, of the 74 chemicals for which there are sufficient concentration data, for 12 of those chemicals there were either five (BDE-100; *p,p′*-DDE; PCBs 153, 170, 187, 201) or six (PCBs 180, 183, 194, 196–203; 1,2,3,4,6,7,8-HpCDD; 1,2,3,4,6,7,8-Hp-CDF) mothers for whom an increase in concentration was seen (*p* = 0.063), although not always for the same mother(s).

#### Milk

**Figure 1:**
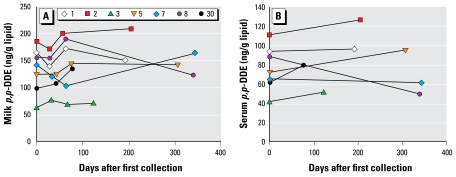
Lipid-adjusted concentrations of DDE (ng/g lipid) over the duration of lactation for the seven participants with first and last collections for both milk and serum. Each line represents an individual.

Milk samples were available from nine of the mothers at 1 and 3 months postpartum. None of the chemicals showed a decrease in concentration in milk for more than six mothers, and none increased in concentration for all nine mothers. For one chemical (*p,p′*-DDE), eight of the mothers (*p* = 0.02) showed an increase in concentration in month 3 compared with month 1. For nine chemicals (BDE-153; *trans*-nonachlor; PCBs 74, 99, 105, 146, 153, 180, 187), seven of the mothers (*p* = 0.09) showed an increase in concentration over the same time frame. Also, not all of the chemicals with increased concentrations from months 1–3 postpartum showed an increase at month 2; thus, the overall direction of the change from month to month is not necessarily consistent. Using a chi-square test for comparing variances, we established that many of the apparent differences between measurements over months 1 and 2 and months 1 and 3 for individual participants cannot be explained by laboratory measurement error. For examples highlighting some of these complexities for DDE and BDE 47, see Figure 1 and Supplemental Material, Figure 2 [available online (doi:10.1289/ehp.0900876.S1)].

### Milk:serum partitioning

**Figure 2:**
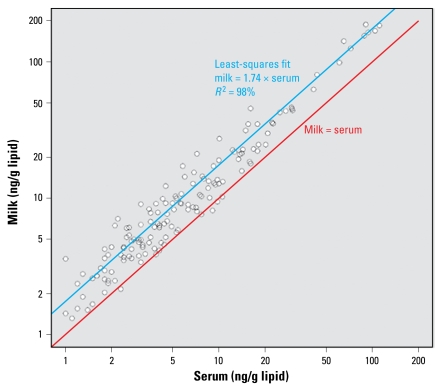
Relationship between lipid-adjusted milk:serum concentrations (ng/g lipid) of two PBDEs (BDE 47, BDE 100); four pesticides/metabolites (HCB, trans-nonachlor, *p,p′*-DDE, *p,p′*-DDT); and nine PCBs (118, 138–158, 146, 153, 170, 180, 187, 74, 99). The red line shows the relationship “serum concentration = milk concentration.” The blue line is the least-squares fit for the data in this study.

For 15 of the chemicals [BDEs 47 and 100; HCB; *trans*-nonachlor; *p,p′*-DDE; *p,p′*-DDT; and nine PCBs (118, 138–158, 146, 153, 170, 180, 187, 74, 99)], the slopes of the lines representing the ratio of lipid-adjusted milk:serum values are not typically equal to 1 as hypothesized and are statistically significantly larger than 1 (Figure 2), although the fitted slopes are within a factor of 3 to the hypothesized 1:1 correspondence [see Supplemental Material, Table 2, available online (doi:10.1289/ehp.0900876.S1)]. The relationship for these chemicals as a group is


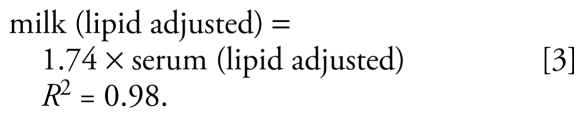


**Figure 3:**
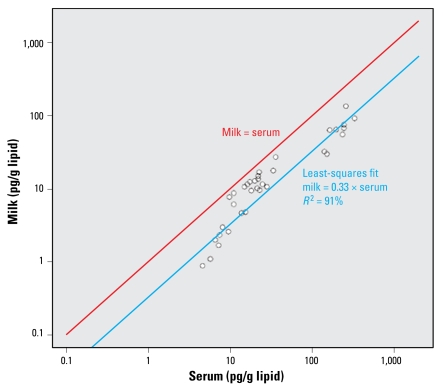
Relationship between lipid-adjusted milk:serum concentrations (pg/g lipid) for three PCDDs (1,2,3,6,7,8-HxCDD, 1,2,3,4,6,7,8-HpCDD, OCDD) and 1,2,3,4,6,7,8-HpCDF. The red line shows the relationship “serum concentration = milk concentration.” The blue line is the least-squares fit for the data in this study.

However, for four chemicals (1,2,3,6,7,8-HxCDD; 1,2,3,4,6,7,8-HpCDD; OCDD; 1,2,3,4,6,7,8-HpCDF), the slopes of the lines representing the ratio of lipid-adjusted milk:serum values were statistically significantly less than 1 (Figure 3). For these chemicals, the fitted slopes are approximately within an order of magnitude of the hypothesized 1:1 correspondence [see Supplemental Material, Table 2, available online (doi:10.1289/ehp.0900876.S1)]. The relationship for these chemicals is


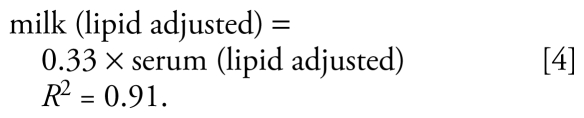


For PCB-126, the slope is not significantly different from 1.0.

The same results were obtained for the slopes for the two sets of chemicals using the log scale approach.

The hypothesis of a consistent relationship between milk and serum concentrations on a lipid-adjusted basis would be undermined should another physicochemical property have a stronger effect than the lipophilicity of the chemical. Linear regression of log *K*_ow_ or molecular weight and slopes of milk:serum relationships gave poor correlations (*R*^2^ = 49%, *R*^2^ = 12%, respectively). However, after separating the chemicals by class (pesticides, dioxin-like compounds, and PCBs) and conducting a similar analysis, it appears that molecular weight explains some of the relationship between chemical levels in milk and serum [see Supplemental Material, Figure 2, available online (doi:10.1289/ehp.0900876.S1)]. Likewise, using this approach, log *K*_ow_ gave similar results in terms of slope versus log *K*_ow_ relationships, but it was not as good a predictor for the pesticides and PCBs (data not shown).

The analysis of the data focused on developing generalizable relationships across classes of persistent, lipophilic chemicals. Degree of chlorination across chemical classes was not significantly associated with milk:serum partitioning. However, for the non-dioxin-like PCBs with at least 50% of the values above LOD, degree of chlorination was strongly associated with partitioning (*R*^2^ = 0.88).

## Discussion and Conclusions

Our goals in this study were to characterize *a*) changes in breast milk concentrations of several groups of persistent organic chemicals during lactation, and *b*) the relationship between lipid-adjusted concentrations of those chemicals in serum and breast milk. Past published research has suggested both a decrease in concentration over the course of lactation and an approximate 1:1 lipid-adjusted milk:serum relationship. However, our results more closely parallel recent studies on depuration of chemicals by lactation that show variable changes in milk concentrations over lactation. Our results also suggest a more complex milk:serum relationship

### Depuration

Our results show that lipid-adjusted concentrations of PBDEs, PCBs, PCDDs/Fs, and organochlorine pesticides in serum and milk do not reliably decrease over the course of lactation and may increase for some women. This is in contrast with a more recent study on depuration of PBDEs and PCBs in milk (Hooper et al. 2007), where a small but consistent decrease in milk concentration (12–18% for PBDEs and 4% for PCBs) was observed over 6 months of breast-feeding.

In our study, breast milk concentrations for many of the chemicals increased between months 1 and 3 postpartum for most mothers. Similarly, serum concentrations increased for many of the chemicals between sampling at 1 month postpartum and end of lactation. However, examination of specific chemicals suggests a more complicated picture. For example, for months 1–3 postpartum, concentrations of *p,p′*-DDE decreased in one mother’s breast milk by 27% but increased in the milk of the other eight mothers by 4–37% (Figure 1). For BDE-153, concentrations in milk decreased over the same time frame in two of the mothers (by 7% and 35 %) but increased in six mothers by 2–116%. Concentrations of PCB-153 decreased in two mothers (3% and 33%) and increased in the others (5–53%). Thus, the trend of the chemical concentration is mother dependent and does not appear to be related to her initial concentration.

Similar complexities in changes in chemical concentration over time were reported by Jin and Su (2005), who observed a decline in average toxic equivalents (TEQs) in milk over the first 30 days of lactation but with concentrations of certain congeners increasing during that time. Miyata et al. (2001) also reported that PCDDs/Fs in milk did not decline in a consistent manner: Some participants’ levels declined whereas others increased postpartum.

This prompts the question: What factors determine whether concentrations increase, decrease, or remain approximately the same over lactation? Two factors, postpartum weight loss and current diet, have been hypothesized and are discussed here in the context of our results.

### Postpartum weight loss

If a mother experiences substantial weight loss postpartum, it has been hypothesized that the remobilization of adipose tissue could produce an increase in circulating levels of previously stored lipophilic chemicals, resulting in an increase (or less of a decrease) in breast milk levels (LaKind 2007; Lovelady et al. 1999). It has also been suggested that mobilization of stored chemicals in adipose tissue can enhance their enterohepatic clearance (Jandacek and Tso 2001).

A limited number of studies have examined the influence of postpartum weight change on environmental chemical concentrations in breast milk (reviewed in Table 3, LaKind 2007). Except for a study of one woman by Vaz et al. (1993), no effect of postpartum weight loss was observed.

In this current study, weight and body mass index (BMI) data were collected at 1 month postpartum but not at the end of the study period. Thus, we cannot evaluate the relationship between change in weight or BMI and concentrations of chemicals in breast milk in the postpartum period. However, if it is hypothesized that weight loss would result in mobilization of adipose tissue and associated lipophilic chemicals, then we should notice consistency in increases in lipid-adjusted levels of highly lipophilic chemicals in the serum and milk compartments for individual women. Our data do not demonstrate this consistency. Focusing on chemicals that are tightly regulated (banned or have strict controls on emissions) and are thought to have similar routes of exposure (principally via diet), it is clear that within one mother, changes (increase vs. decrease) in concentrations of these chemicals over time are not consistent. For example, whereas one participant (no. 30) showed increases in all chemicals (including PBDEs) for which levels were above the detection limit for both sampling periods (1 and 3 months postpartum for milk and serum), for the other participants, some chemical concentrations increased while other decreased during the same period. Participant no. 5’s serum and milk lipid-adjusted concentrations of 1,2,3,4,6,7,8-Hp-CDD increased over lactation, but serum and milk OCDD decreased. The direction of change in concentration was matrix dependent for HCB, PCB-170, PCB-126, and 1,2,3,4,6,7,8-HpCDF. In another participant (no. 7), serum levels for most of the chemicals decreased, but again, several of the milk concentrations increased, and several chemicals did not change in a consistent direction for both matrices. Perhaps the most inconsistent was participant no. 8, whose serum levels decreased for almost all of the chemicals studied (from 1 to 12 months postpartum), but whose breast milk levels increased (from months 1 to 3 postpartum) for most of those chemicals. Other participants had similar incongruities in their serum and milk data.

The lack of consistency in increase or decrease in concentrations across chemicals and matrices makes it less likely that weight change is a dominant contributing factor to levels of chemicals in breast milk, but it does suggest that the controlling factors are likely numerous and complex.

### Diet

Dietary lipids are thought to contribute approximately 29% of breast milk lipid (Hachey et al. 1987), and for many persistent organic compounds, diet is considered the principal route of exposure. (The exception may be the PBDEs, for which diet and household dust are both thought to be significant contributors.) It has been assumed that because many of the persistent organic chemicals are legacy chemicals (that is, they are no longer in production and their current releases are negligible), levels in women today can be attributed principally to lifetime accumulation. This implies that there are no actions that breast-feeding women can take to reduce their breast-fed infants’ exposures. However, recent research on within-day variability in dioxin breast milk concentrations and correlation with diet and fasting suggests that diet during lactation may play an important role in influencing levels of persistent organic compounds in breast milk (Miyata et al. 2001, 2006). Specifically, breast milk (30 samples over 5 days) and samples of diet (over 3 days) were obtained from one mother, and breast milk from two additional mothers was obtained over 5 days, 3 days of which the two mothers fasted (Miyata et al. 2006). Levels of dioxin TEQs in the first mother’s diet and her breast milk exhibited large fluctuations over the 3 days. Dioxins in breast milk also decreased substantially during fasting. The short time frame over which the Miyata et al. (2006) research was conducted further suggests that diet, rather than weight change, influenced levels in milk. The plausibility of current intake of one group of chemicals (PCDDs/Fs) via current diet being associated with levels in breast milk has been described (LaKind 2007). In this study, we did not measure dietary levels of the chemicals of interest and so cannot form conclusions regarding dietary intake. However, the data showing variations, including increases, in chemical concentrations over lactation do not rule out a dietary influence.

### Milk:serum partitioning

Should consistent, stable, lipid-based, chemical-specific milk:serum partitioning factors be determined, these could allow for the use of serum measurements for estimating levels in milk and for estimating infant exposures from breast-feeding. Some studies have found lipid-adjusted levels of PCDDs, PCDFs, and PCBs to be approximately equivalent in serum and milk [reviewed by Aylward et al. (2003)]. However, other studies have found more complex relationships. For example, Wittsiepe et al. (2004) reported that some PCDDs/Fs and PCBs preferentially partition into lipids of one matrix over another. Factors that may influence partitioning of these compounds include differences in lipophilicity and molecular size and weight, which can affect their membrane permeability (LaKind 2007; Wittsiepe et al. 2004) and degree of chlorination (Patterson et al. 1989). Suzuki et al. (2005) reported relative concentrations of lipid-adjusted PCDD TEQs in the following maternal compartments: placenta > maternal blood > breast milk > adipose tissue > cord blood. For lipid-adjusted PCDFs, a different order was observed: placenta > maternal blood > cord blood > breast milk > adipose tissue. The authors suggest that different chemical groups may have different affinities for specific tissue types. A statistically significant correlation was found between concentrations in adipose tissue and breast milk for the sum of seven PBDEs, but with congener-specific differences (Antignac et al. 2009), for example, for BDEs 99 and 153. Jaraczewska et al. (2006) did not find significant correlations between lipid-adjusted breast milk and maternal serum concentrations for organo-chlorine pesticides (HCB, *p,p′*-DDE, *p,p′*-DDT) or PCBs (118, 138, 153, 170, 180) in 22 women. However, reanalyzing their data excluding one participant whose values were substantially higher than those of the other participants, we find milk:serum relationships from their study consistent with our results for PCBs 138, 153, 170, 180, and *p,p′*-DDE.

Our results showed no clear relationship between log *K*_ow_ and lipid-adjusted milk:serum ratios for the 17 chemicals with sufficient data above the LOD and for which *K*_ow_ values are available. However, the determination of *K*_ow_ for extremely lipophilic chemicals is difficult, and reported results can cover a large range of values. In fact, for many of the chemicals in this study, *K*_ow_ values spanning more than one order of magnitude were found in the published literature.

Within the group of chemicals with milk:serum ratios < 1 (the PCDDs/Fs), there appears to be preferential partitioning into milk for lower weight and smaller *K*_ow_ chemicals. Within the group of chemicals with milk:serum ratios > 1, milk:serum ratios for PCBs were inversely correlated with molecular weight, whereas organochlorine pesticides were positively correlated with molecular weight. A correlation was observed between degree of chlorination and milk:serum ratios for PCBs, with the lower chlorinated PCBs preferentially partitioning into milk, as was observed by Wittsiepe et al. (2007).

Three important conclusions from the results of this study, and supported by previous research, can be drawn:

- Our results suggest that elimination of chemicals via lactation is complex and that decreases in concentrations of lipophilic chemicals over the course of lactation should no longer be assumed. This is true for all of the chemical classes assessed, including PCDDs/Fs, PCBs, organochlorine pesticides, and PBDEs. Because no decrease in concentration over time can be assumed, the concept of pumping and discarding early milk (or “pump and dump”) for breast-feeding mothers as a means of reducing infant exposure (Hooper et al. 2007; Steingraber 2002) is not supported. This conclusion also has important implications for the conduct of research on infant exposure and risk assessments, where declines in breast milk concentrations of these chemicals over lactation have been assumed. Because some of the percent changes during lactation were > 300%, falling outside the range of what would be considered minimal change, studies that incorporate analyses of diet and dust, as well as weight changes over lactation, are needed to better parse out the various factors that may influence chemical concentrations in breast milk over lactation.
- If current diet is a significant source of persistent environmental chemicals in breast milk, this implies that new mothers may be able to take actions that could reduce infant exposure. However, this is a complex issue, as one food group that may contain higher levels of one class of the persistent chemicals (e.g., PCBs in fish) might be replaced with another food group containing other chemicals that may be transferred to breast milk (e.g., dioxins in dairy products). Further study focusing on measurements of chemicals in diet and changes in milk concentration is warranted before specific dietary advice can be given.
- The hypothesis that concentrations of persistent lipophilic chemicals on a lipid-adjusted basis would have consistent relationships across matrices is likely too simplistic. This study revealed two distinct milk:serum ratios, one > 1, and one < 1. Whether these differences would have a substantial impact on infant exposure assessments is less clear. At present, we suggest that the milk:serum ratios given in this paper be used to evaluate infant exposure if only serum data are available. We further recommend that additional studies that include a larger cohort be conducted to confirm these results. Additional components of future research that might elucidate these findings include repeated measures of BMI and assessment of post-pregnancy changes in size of body compartments such as fat and muscle.

## Figures and Tables

**Table 1:** Timing of collection of milk and blood samples.

Participant no.	First collection (date) [infant age, days]	Second collection (days after first collection)	Third collection (days after second collection)	Fourth collection (days after third collection)
30	8/17/04 – M,S [35]	41 days – M	36 days – M,S	–
1	9/13/04 – M,S [37]	28 days – M	35 days – M	129 days – M,S
2	11/30/04 – M,S [34]	28 days – M	28 days – M	149 days – M,S
3	12/27/04 – M,S [30]	32 days – M	35 days – M	56 days – M,S
4^a^	1/25/05 – M,S [31]	–	–	–
5	1/20/05 – M,S [31]	42 days – M	33 days – M	232 days – M,S
7	6/30/05 – M,S [31]	33 days – M	31 days – M	280 days – M,S
8^b^	7/26/05 – M,S [29]	29 days – M	34 days – M	277 days – M,S
9^c^	8/19/05 – M,S [33]	39 days – M	29 days – M	266 days – M
10^d^	10/13/05 – M,S [30]	35 days – M	35 days – M	–

Abbreviations: M, milk; S, serum.

a One collection only because of illness.

b Information available for month and year only.

c Unable to draw blood at end of study.

d Lost contact at end of study.

**Table 2:** Median concentrations in milk and serum lipids and percent detects for all chemicals detected in at least 50% of serum and milk samples.

Chemical	Median (ng/g lipid milk)	No. (milk)	% Detect milk	Median (ng/g lipid serum)	No. (serum)	% Detect serum
BDE-047	26	35	100	15.5	17	100
BDE-100	4	35	100	2.4	17	100
BDE-153	3.5	35	100	4	17	58.8
HCB	8.05	35	100	6.9	17	88.2
Oxychlordane	13.65	35	94.3	5.1	17	82.4
*t*-Nonachlor	14.5	35	100	5.8	17	94.1
*p*,*p′*-DDE	142	35	100	79.3	17	100
*p*,*p′*-DDT	5.46	35	97.1	2.5	17	94.1
Mirex	0.84	35	91.4	0.8	17	76.5
PCB-105	1.65	35	100	0.8	17	88.2
PCB-118	6.51	35	100	4	17	100
PCB-138-158	20.47	35	100	13.2	17	100
PCB-146	2.4	35	97.1	1.7	17	100
PCB-153	22.85	35	100	15.9	17	100
PCB-156	3	35	97.1	2.2	17	70.6
PCB-170	4.28	35	100	3.9	17	100
PCB-177	0.86	35	77.1	0.6	17	52.9
PCB-178	0.97	35	85.7	0.8	17	70.6
PCB-180	10.49	35	100	9.8	17	100
PCB-183	1.55	34	88.2	1.2	17	94.1
PCB-187	3.61	35	97.1	2.7	17	100
PCB-194	1.46	35	85.7	1.7	17	94.1
PCB-196-203	1.63	35	94.3	1.9	17	94.1
PCB-201	1.49	35	88.6	1.7	17	88.2
PCB-206	0.5	35	80	1	17	88.2
PCB-28	2.12	35	65.7	0.9	17	58.8
PCB-66	0.95	34	94.1	0.6	17	58.8
PCB-74	6.4	35	100	3.5	17	100
PCB-99	5.47	35	100	3.1	17	100
Chemical	Median (pg/g lipid milk)	No. (milk)	% Detect milk	Median (pg/g lipid serum)	No. (serum)	% Detect serum

1,2,3,6,7,8-HxCDD	11.40	33	100	15.00	17	100
1,2,3,4,6,7,8-HpCDD	10.87	34	100	22.60	17	100
OCDD	70.00	34	100	211.0	17	100
2,3,4,7,8-PnCDF	3.81	34	88.2	4.20	17	94.1
1,2,3,4,7,8-HxCDF	1.92	33	93.9	3.30	17	64.7
1,2,3,6,7,8-HxCDF	1.63	33	72.7	3.60	17	76.5
1,2,3,4,6,7,8-HpCDF	2.32	34	97.1	7.40	17	100
OCDF	0.17	34	50	5.30	17	94.1
PCB-126	12.19	34	100	12.00	17	100
PCB-169	7.36	34	100	8.10	17	94.1

Abbreviations: HxCDF, hexachlorodibenzofuran; OCDF, octachlorodibenzofuran; PnCDF, pentachlorodibenzofuran.
